# Sub-terahertz silicon-based on-chip absorption spectroscopy using thin-film model for biological applications

**DOI:** 10.1038/s41598-022-21015-8

**Published:** 2022-10-22

**Authors:** Seyed Ali Hosseini Farahabadi, Milad Entezami, Hesam Abouali, Hadi Amarloo, Mahla Poudineh, Safieddin Safavi-Naeini

**Affiliations:** 1 Centre for Intelligent Antenna and Radio Systems (CIARS), Department of Electrical and Computer Engineering, University of Waterloo, Waterloo, N2L 3G1 Canada; 2 Department of Electrical and Computer Engineering, University of Waterloo, Waterloo, N2L 3G1 Canada

**Keywords:** Electrical and electronic engineering, Biomedical engineering, Nanobiotechnology

## Abstract

Spectroscopy in the sub-terahertz (sub-THz) range of frequencies has been utilized to study the picosecond dynamics and interaction of biomolecules. However, widely used free-space THz spectrometers are typically limited in their functionality due to low signal-to-noise ratio and complex setup. On-chip spectrometers can revolutionize THz spectroscopy allowing integration, compactness, and low-cost fabrication. In this paper, a low-loss silicon-based platform is proposed for on-chip sub-THz spectroscopy. Through functionalization of silicon chip and immobilization of bio-particles, we demonstrate the ability to characterize low-loss nano-scale biomolecules across the G-band (0.14–0.22 THz). We also introduce an electromagnetic thin-film model to account for the loading effect of the immobilized biomolecules, i.e. dehydrated streptavidin and immunoglobulin antibody, as two key molecules in the biosensing discipline. The proposed platform was fabricated using a single mask micro-fabrication process, and then measured by a vector network analyzer (VNA), which offers high dynamic range and high spectral resolution measurements. The proposed planar platform is general and paves the way towards low-loss, cost-effective and integrated sub-THz biosensors for the detection and characterization of biomolecules.

## Introduction

Sub-terahertz (sub-THz) frequencies—referring to 0.1–0.5 THz—have been under a great deal of research and development. Due to the unique features of THz wave such as non-ionizing photon energy, high penetration through optically non-transparent material and unique spectral fingerprint of large biomolecules, it has found numerous applications in the field of imaging, communication, radio astronomy, and sensing^1–4^. In particular, molecular biology benefits from sub-THz spectroscopy to study bio-molecular dynamics occurring on nanosecond to femtosecond timescales^5,6^. Time-domain free-space absorption spectroscopy has become a mature and ubiquitous technology to analyze bio-samples^7–9^. However, the strong absorption of THz wave in aqueous samples has made the free-space measurements fairly challenging and necessitated to decrease the interaction volume of the sample and THz signal, which avoids a large attenuation of THz wave. Consequently, the low interaction length of the wave and bio-particles in the sample results in less sensitive measurements. It is worth noting that most of the commercially available bio-samples are usually expensive and prepared in small volumes. Indeed, this has already restricted the available volume of the bio-sample under test. Although the time-domain spectroscopy (TDS) is a a broadband technique with a relatively large dynamic range, it has a relatively small signal-to noise-ratio (SNR)^10^. The limited SNR would largely affect the limit of detection, which makes it hard to analyze low concentration biosamples. Furthermore, the free-space setup is essentially complex and needs bulk quasi-optical elements such as lenses and mirrors to control and focus the beam on the sample.

Time-domain on-chip spectroscopy provides a potential solution to overcome some of the challenges of free-space methods^11–13^. Waveguide plays an essential role in the sensing and characterization process. This role largely depends on the interaction of waveguide mode, or guided electromagnetic wave (EM), with the biosample. Increasing the waveguide length, even up to a few centimeters, could result in more effective interaction. Furthermore, wave propagation within the waveguide offers better SNR. Waveguide can also be engineered to detect and characterize nano-scale bio-molecules; consequently, a lower sample is required for the measurement. Over the past few years, on-chip devices have utilized different types of metallic waveguides such as single wire waveguide^13^, microstrip line^11,12^, and coplanar waveguide^14^. Conduction loss of metals drastically increases at sub-THz and THz frequencies, making the characterization of low-loss biomolecules challenging. Moreover, the fabrication of these on-chip devices is not necessarily cheap and simple, as it involves multiple materials with different fabrication processes. It is worth mentioning that the free-space coupled waveguides still suffer from the system complexity, as the optical components require careful alignment. Therefore, a miniaturized platform compatible with an integrated system is demanding.

In this paper, a silicon-based chip is proposed to characterize biomolecules in the sub-THz range of frequencies. Waveguides made of high-resistivity silicon (HR-Si) incurs lower insertion loss, and as a result, waveguide length can be extended to achieve better wave-mater interaction^15–20^. In contrast to a photonic crystal waveguide in which guiding channel is supported by in-plane band-gap structures, all sides of the proposed suspended silicon waveguide are accessible and surrounded by air. Therefore, the number of immobilized biomolecules on the surface of the waveguide gets increased, leading to the more interaction of electromagnetic wave and matter. Moreover, the suspended strip waveguide can be used over a wider bandwidth compared to photonic crystal waveguide. Well-developed fabrication processes of silicon technology enable the high-precision making of low-cost bio-devices. This work utilizes a vector network analyzer (VNA) to carry out frequency-domain spectroscopy by measuring scattering parameters of the waveguide as a two-port network. The VNA works based on the electronic heterodyne technique and provides high-frequency resolution (down to $\sim \,100$ Hz) and relatively large dynamic range. We show that the biomolecules can be modeled by a surface admittance, a more reasonable model for nano-scale biomolecules rather than a thin bulk slab.

This article is structured as follows. At first, the silicon waveguide’s theory and design are described. Next, the thin-film model is demonstrated, and the electromagnetic modeling of biomolecules is discussed. Finally, the fabrication process, immobilization of biomolecules on the silicon platform and measurement results are discussed.

**Figure 1:**
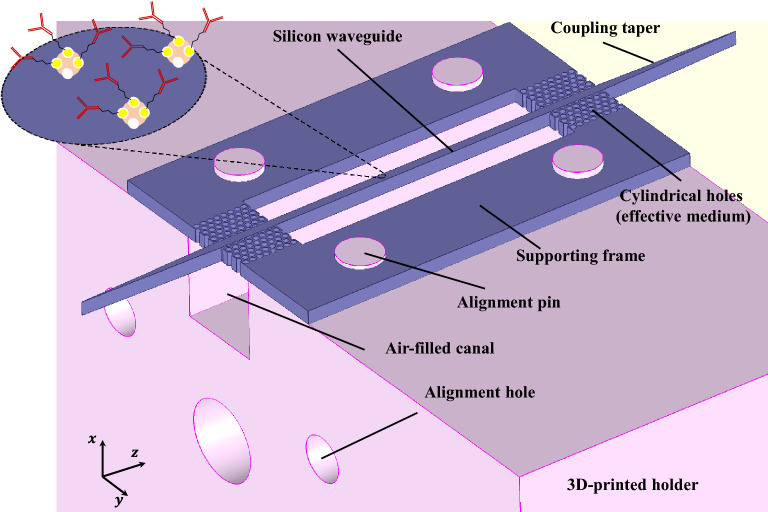
Schematic illustration of silicon-based platform for biomolecule characterization. Silicon chip  (purple color) includes a straight waveguide supported by a frame through condensed lattices of cylindrical holes (effective medium), which avoid power leakage in the frame. The surface of the waveguide is functionalized with proper receptors to immobilize the desired particles. The biomolecules are characterized using additional loss as compared to the unloaded waveguide. Silicon chip is fixed on a 3D-printed holder (pink color) to reduce the misalignment during the measurement.

## Silicon waveguide design

The general configuration of the platform is shown in Fig. 1. It consists of a straight waveguide and a supporting frame made of HR-Si (purple color). The surface of the waveguide can be treated for desired biomolecules immobilization. The trapped particles show an additional loss compared with the unloaded waveguide. The intersection points of the waveguide and frame are perforated with cylindrical holes to avoid power leakage to the frame. In fact, the lattice realizes an effective medium with low permittivity such that the waveguide mode remains approximately confined while passing through it. Two ends of the waveguide are progressively tapered to match the particular mode of the dielectric waveguide to that of metallic ones, which will be used in the measurement setup. By this means, the possible reflections and scatterings are removed. The silicon frame is fixed on a 3D-printed structure (pink color) using four alignment pins to reduce the number of uncertainties during the measurement.

**Figure 2:**
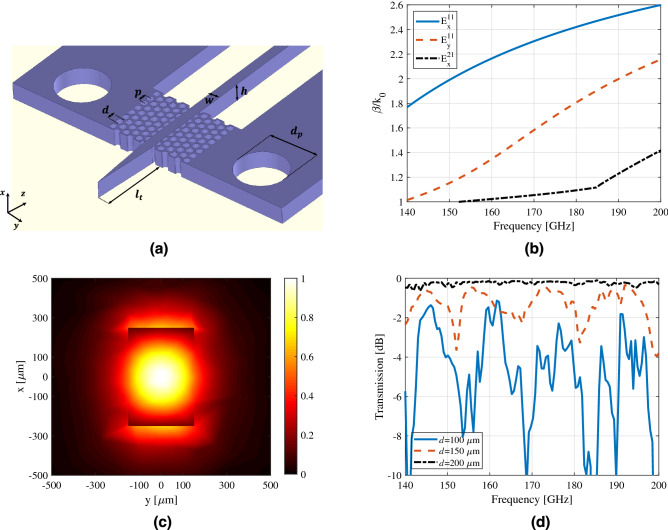
(**a**) Illustration of physical parameters of HR-Si waveguide [$w=300\,\upmu$m,$\,h=500\,\upmu$m,  $d=200\,\upmu$m, $p=250\,\upmu$m, $l_{t}=5$ mm, $d_{p}=1.5$ mm]. (**b**) Dispersion diagram of the first three potential guided modes through the silicon waveguide. (**c**) Normalized electric field distribution of the waveguide’s fundamental mode $E_{x}^{11}$ at 170 GHz. (**d**) Parameter study of insertion loss for the waveguide with a length of 24 mm. Changing the diameter of holes *d* in the lattice structure affects the loss such that larger d results in smaller insertion loss.

### Modal analysis

Silicon waveguide is capable of supporting quasi-TE and -TM modes. Similar to metallic waveguides, physical parameters of a dielectric waveguide such as height (*h*), width (*w*) and constituent material ($\varepsilon _{r}$) determine the modal behavior (Fig. 2a). Commercially available High Frequency Structure Simulator (HFSS) is used to carry out modal analysis. In this study, relative permittivity $\varepsilon _{r}=11.7$ and $\sigma =0.01$ s/m are assumed for the silicon modeling^15^. Figure 2b shows the dispersion diagram for the first three modes of the silicon waveguide with $w=300\,\upmu$m and $h=500\,\upmu$m over 140–200 GHz. These parameters are set in such a way that the waveguide supports $E^{x}_{11}$, out of plane polarized mode. Due to the x-polarized excitation, the second mode ($E_{y}^{11}$) is not propagated in the waveguide. Moreover, the odd symmetry of the third mode ($E_{x}^{21}$) is not matched to the even symmetrical source used as the excitation. Hence, it is weekly propagative. The normalized field distribution of the fundamental mode ($E_x^{11}$) is depicted in Fig. 2c. Owing to the larger permittivity of the silicon compared to the air, the electric field is properly confined inside the waveguide. Interestingly, the continuity of the normal component of electric flux mandates a relatively strong electric field at two horizontal boundaries of the waveguide. These two sides of the waveguide are much more sensitive to the loading effect of bioparticles.

At the intersection region of the silicon frame and the waveguide, cylindrical holes are perforated to reduce the effective permittivity and remove the leakage and possible scatterings inside the waveguide. These holes are located in an equilateral triangular lattice^21^ with the optimized pitch size $p=250\,\upmu$m and hole diameter $d=200\,\upmu$m. It is noted that by decreasing the hole’s diameter, the insertion loss increases, which refers to the power leaked to the frame (Fig. 2d). On the other hand, considering the mechanical stability of the silicon waveguide, the diameter of holes cannot be increased beyond the optimized value.

## Electromagnetic modeling of biomolecules

We assumed that loading bio-molecules on the dielectric waveguide forms an effective monolayer of particles on all sides of that. This reasonable assumption originates from the fact that the distances between randomly distributed bio-particles on all four waveguide boundaries are much smaller than the guided wavelength. This monolayer interacts with the guided mode, leading to a change in the waveguide insertion loss. Size of the bio-particles, and inter-molecular interactions are significant factors contributing to the loss level. It is common to model the bio-particles with an effective thin dielectric using bulk parameters, whereas this type of modeling can easily fail^22^. The size of immobilized bioparticles on all sides of the waveguide is usually much smaller than the guided wavelength. So, modeling them with an effective dielectric slab with finite thickness is not necessarily a proper choice, particularly when bio-molecules are characterized without any background solution. Moreover, the slab thickness can not be chosen uniquely. It can be different for a specific bio-molecule from one model to another one. Even in the case of using the slab model and reducing the slab thickness to the actual size of the bio-particles in EM simulators, the minimum computational mesh size should be determined so that at least one cell of the mesh falls within the slab; however, the size of the whole chip could be much larger than the guided wavelength. Dealing with such a multi-scale problem increases the required computational effort^22^.

We propose using an effective surface admittance boundary condition to model bio-particles and overcome the aforementioned constraints. In this method, particles are generally replaced by an equivalent admittance sheet which is defined as^23^

1 $$\begin{aligned} \hat{n}\times \mathbf{H} _{t}=\mathbf{J} _{t}=Y_{s} \mathbf{E} _{t} \end{aligned}$$

where $Y_{s}=g_{s}+jb_{s}$ denotes complex surface admittance, $\hat{n}$ is the unit normal vector to the surface of the waveguide, $\mathbf{J} _{t}$ is the surface current vector, $\mathbf{H} _{t}$ and $\mathbf{E} _{t}$ are the tangential magnetic and electric fields at the surface of the waveguide, respectively. Leveraging this model, biomolecules are treated as a sheet of current or simply a boundary condition, which removes the ambiguity of choosing the thickness of the slab in the bulk model.

**Figure 3:**
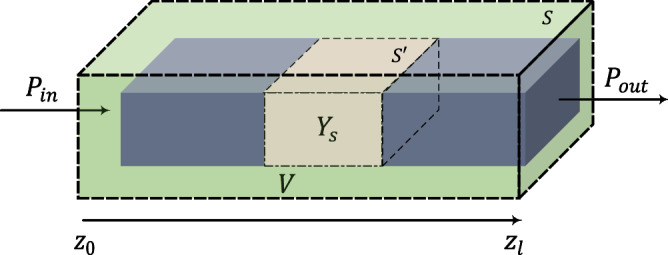
Schematic diagram of the silicon waveguide partially loaded ($S^{'}$) by the surface admittance ($Y_{s}$). Poynting’s theorem is utilized to extract the admittance of the thin film-model in terms of input and output power.

Now, Poynting’s theorem is utilized to derive the admittance in the proposed thin-film model, where a standalone waveguide partially loaded with the equivalent surface admittance is considered (Fig. 3). According to Poynting’s theorem over the volume V enclosed with surface S,

2 $$\begin{aligned} P_{s}=P_{e}+P_{d}+j2\omega (\overline{W}_{m}-\overline{W}_{e}) \end{aligned}$$

where $P_{s}$ is the supplied complex power inside V, $P_{e}$ is the complex power exiting the volume obtained by

3 $$\begin{aligned} P_{e}=\frac{1}{2}\int _{S}(\mathbf{E} \times \mathbf{H} ^{*})\cdot \mathbf{ds} , \end{aligned}$$

$P_{d}$ is the complex dissipated power over volume V,

4 $$\begin{aligned} P_{d}=\frac{1}{2}\int _{V}(\mathbf{E} \cdot \mathbf{J} ^{*})dv, \end{aligned}$$

and $\overline{W}_{m}$ and $\overline{W}_{e}$ are time average magnetic and electric energy stored in volume V. In our case, as there is no impressed source inside volume V, $P_{s}$ vanishes. Interestingly, $\overline{W}_{m}$ and $\overline{W}_{e}$ are equal due to the general property of waveguiding system^24^. Therefore, equation (2) becomes

5 $$\begin{aligned} P_{e}+P_{d}=0. \end{aligned}$$

Knowing that for a well-designed waveguide, fields are largely confined inside the waveguide, surface integral in Eq. (3) can be written as

6 $$\begin{aligned} P_{e}=-\frac{1}{2}\int _{s_{z_{0}}}(\mathbf{E} \times \mathbf{H} ^{*})\cdot \hat{z}ds+\frac{1}{2}\int _{s_{z_{l}}}(\mathbf{E} \times \mathbf{H} ^{*})\cdot \hat{z} ds \end{aligned}$$

where $s_{z_{0}}$ and $s_{z_{l}}$ are the cross-sections of the waveguide at $z_{0}$ and $z_{l}$, respectively. In the case of the loaded waveguide, silicon loss and bio-film absorption are two critical factors. To derive the dissipated power in bio-film, it is assumed that the electromagnetic fields inside the loaded waveguide do not change significantly. This assumption allows using perturbation theorem, which facilitates the loss calculation^25^. As such, the total loss can be calculated using Eqs. (1)–(4)

7 $$\begin{aligned} P_{d}=P_{d}^{Si}+P_{d}^{film} \end{aligned}$$

where

8 $$\begin{aligned}&P_{d}^{Si}=\frac{1}{2}\sigma _{Si}\int _{v}|\mathbf{E} |^{2}dv \end{aligned}$$

9 $$P_{d}^{film}=\frac{1}{2}Y_{s}\int _{S'}|\mathbf{E} _{t}|^{2}ds^{\prime}$$

in which $\sigma _{Si}$ denotes silicon bulk conductivity and $\mathbf{E} _{t}$ is the tangential component of the unperturbed electric field on the waveguide. Accordingly, the real and imaginary part of admittance can be derived as

10 $$g_{s} = \Re \{ Y_{s} \} = \frac{{\int_{{s_{{z_{0} }} }} \Re \{ {\mathbf{E}} \times {\mathbf{H}}^{*} \} \cdot \hat{z}ds - \int_{{s_{{z_{l} }} }} \Re \{ {\mathbf{E}} \times {\mathbf{H}}^{*} \} \cdot \hat{z}ds - \sigma _{{Si}} \int_{V} | {\mathbf{E}}|^{2} dv}}{{\int_{{S^{\prime}}} | {\mathbf{E}}_{t} |^{2} ds^{\prime}}}{\text{ }},{\text{ }}$$

11 $$b_{s} = \Im \{ Y_{s} \} = \frac{{\int_{{s_{{z_{0} }} }} \Im \{ {\mathbf{E}} \times {\mathbf{H}}^{*} \} \cdot \hat{z}ds - \int_{{s_{{z_{l} }} }} \Im \{ {\mathbf{E}} \times {\mathbf{H}}^{*} \} \cdot \hat{z}ds}}{{\int_{{S^{\prime}}} | {\mathbf{E}}_{t} |^{2} ds^{\prime}}}.{\text{ }}$$

Later on, in the measurement results, it is shown how to calculate the $g_{s}$ based on measured scattering parameters of the waveguide.

## Fabrication

Waveguides were fabricated from an intrinsic HR-Si wafer, employing photolithography and deep reactive ion etching (DRIE). DRIE is a highly anisotropic etching process and used to create features with high aspect ratio. The Bosch process is widely used to make hollows with almost vertical sidewalls.

The first fabrication step was to spin-coat the silicon wafer with a thick photoresist (AZ P4620), almost $11\,\upmu$m. Then, the wafer was soft-baked at $90\,^{\circ }$C for 115 s. Rehydration process time was considered 3 h. Afterward, the photoresist was patterned with the waveguide structure by employing optical lithography. Eventually, the etching process was carried out by using the DRIE to form the whole platform. Based on what has been explained in^15^, this single mask process leads to high precision devices.

The fabricated waveguides can be directly used for measurement. However, the alignment issue is quite challenging to achieve repeatable and accurate results. To mitigate this problem, a 3D-supporting structure was designed and printed using Raise3D Pro2 printer. This structure includes four alignment pins that pass through the four holes on the waveguide frame, as shown in Fig. 1. Moreover, a rectangular canal was removed from the printed shell below the silicon waveguide to avoid any possible interaction. Two holes on the end sides of the shell were utilized to align the metallic waveguide flange and the device under the test.

**Figure 4:**
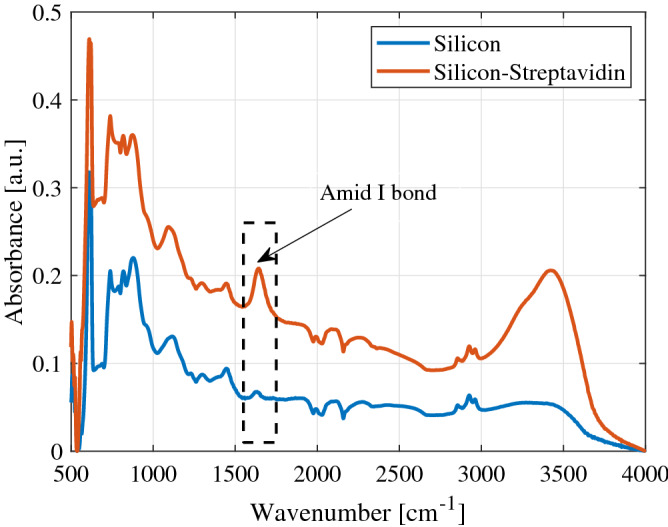
Infrared spectra of bare silicon sample and silicon treated by streptavidin. Observation shows a significant peak at 1650 cm$^{-1}$ due to the formation of Amide I bond between the carboxyl groups of the substrate and amine groups in the streptavidin protein.

## Sample preparation

Proteins can be attached to solid substrates through different physisorption and chemisorption approaches^26^. The physical adsorption of biomolecules such as streptavidin has been studied on polystyrene^27^ and silicon^28^ substrates. Since the physisorption does not need any complex multi-step surface modifications, a physically-based attachment of streptavidin has been implemented for the first characterization of the developed platform. The streptavidin-coated substrates were prepared through physisorption of streptavidin on the cleaned silicon. The silicon substrates were cleaned with acetone following by isopropyl alcohol (IPA) and plasma treatment. Next, $50 \upmu$g/mL of streptavidin in phosphate-buffered saline (PBS) buffer was added to the substrates, and incubation was done at room temperature overnight. Excess streptavidin was removed, and the substrates were washed with PBS buffer. The successful adsorption of the mentioned proteins has been verified by attenuated total reflectance-Fourier transform infrared (ATR-FTIR) spectroscopy. After modification of the substrates with streptavidin, a peak at 1650 cm$^{-1}$ wavenumber, and a subsequent decrease in absorbance after this peak can be observed in Fig. 4. This peak is associated with the presence of Amide I bond between the carboxyl groups of the substrate and amine groups in the streptavidin protein.

**Figure 5:**
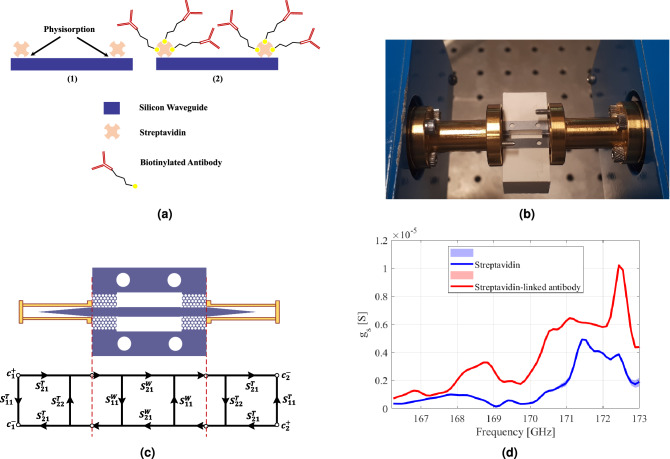
(**a**) Schematic illustration of prepared bio-samples. In (1) the streptavidin molecules are physisorbed on the surface of the silicon waveguide while in (2), Ig-G antibodies are immobilized on the silicon treated by streptavidin molecules. (**b**) Measurement set-up for characterization of biomolecules. For excitation, two tips of silicon waveguide are inserted inside of the conventional metallic waveguides. (**c**) Signal flow graph of the measurement set-up (**d**) Measured surface conductivity $g_{s}$ of streptavidin and Ig-G adsorbed on streptavidin using the thin-film model.

The lack of this peak in the non-modified silicon substrate is evident and in agreement with previous studies^29,30^. The other sample analyzed in this study was the anti-human Ig-G antibody. First, the mentioned antibody was biotinylated and concentrated to 10 $\upmu$g/mL. Then, it was incubated with the previously streptavidin-coated substrates overnight at 4 $^{\circ }$C. After removal of the antibodies, the substrates were washed by PBS buffer and dried with gentle nitrogen gas. Figure 5a depicts the prepared biosamples for the measurement.

## Measurement results

The measurement setup is shown in Fig. 5b. The insertion loss of the fabricated devices was measured using PNA-X (Keysight N5242A) connected to two OML WR05 frequency extenders. To couple power from standard rectangular waveguides to the proposed dielectric waveguide, tapered sections of the silicon channel are placed inside the rectangular waveguide sections. Three waveguide sets were prepared for the measurements, unloaded waveguide as the reference, waveguide coated with streptavidin itself, and one with Ig-G antibodies captured by the streptavidin.

The first step to extracting the effect of bioparticles is to deembed the taper sections at two ends of the waveguide. Figure 5c shows the signal flow graph of the measurement system from port 1 to port 2. In this figure, $c_{1}^{+}$ and $c_{2}^{+}$ are the incident wave amplitudes, and $c_{1}^{-}$ and $c_{2}^{-}$ are the reflected wave amplitudes. The two transition parts are assumed to be the same and described by $[S^{T}]$, while the waveguide section is shown by $[S^{W}]$. As the waveguide is passive, symmetric and reciprocal, $S^{W}_{11}=S^{W}_{22}$ and $S^{W}_{21}=S^{W}_{21}$. Given signal flow, $S_{21}$ can be derived as

12 $$S_{{21}} = \frac{{c_{2}^{ - } }}{{c_{1}^{ + } }}\bigg |_{{c_{2}^{ + } = 0}} = \frac{{(S_{{21}}^{T} )^{2} S_{{21}}^{W} }}{{\left( {1 - S_{{11}}^{W} S_{{22}}^{T} } \right)^{2} - \left( {S_{{22}}^{T} S_{{21}}^{W} } \right)^{2} }}.{\text{ }}$$

As the tapers are designed to match the metallic waveguide mode to that of dielectric one, $S^{T}_{22}$ is negligible. Hence, $S_{21}$ is simplified as

13 $$\begin{aligned} S_{21}\bigg |_{S^{T}_{22}=0}=(S^{T}_{21})^2S^{W}_{21}. \end{aligned}$$

Equation (13) relates the measured $S_{21}$ by the VNA to the $S_{21}^{W}$, corresponding to that part of the waveguide, which is functionalized by the biomolecules. Deriving the same equation for the case of the unloaded waveguide and dividing that by Eq. (13) leads to

14 $$\begin{aligned} \frac{S_{21}^{M:Ref}}{S_{21}^{M,Loaded}}=\frac{S_{21}^{W:Ref}}{S_{21}^{W:Loaded}}. \end{aligned}$$

As the bio-particles are extremely small and analyzed in a dehydrated condition, they are not able to change the propagation constant of the silicon waveguide. Therefore, we assume that $y_{s}\approx 0$ and bio-molecules are modeled by a purely resistive sheet. knowing that

15 $$\frac{{\int_{{s_{{z_{0} }} }} \Re \{ {\mathbf{E}} \times {\mathbf{H}}^{*} \} \cdot \hat{z}ds - \int_{{s_{{z_{l} }} }} \Re \{ {\mathbf{E}} \times {\mathbf{H}}^{*} \} \cdot \hat{z}ds}}{{\int_{{s_{{z_{0} }} }} \Re \{ {\mathbf{E}} \times {\mathbf{H}}^{*} \} \cdot \hat{z}ds}} = 1 - |S_{{21}}^{W} |^{2} ,$$

combining Eqs. (10) and (14) gives the $g_{s}$ as

16 $$g_{s} = \left( {\frac{{1 - |S_{{21}}^{{M:loaded}} |^{2} }}{{1 - |S_{{21}}^{{M:Ref}} |^{2} }} - 1} \right)\frac{{\sigma _{{Si}} \int_{V} | E|^{2} dv}}{{\int_{{S^{\prime}}} | E_{t} |^{2} ds^{\prime}}}.{\text{ }}$$

Figure 5d illustrates the surface conductance $g_{s}$ for both cases of streptavidin and streptavidin-linked antibody immobilized on the surface of the waveguide. Interestingly, both conductances are frequency dependant while the latter one introduces more loss on the waveguide. These measurements were carried out multiple times to ensure that the results were repeatable. The variation of the derived conductance in the proposed thin-film model shown in Fig. 5d also verifies this statement. Employing this model, a collective and macroscopic view of bio-molecules can be obtained, which unveils the nano-scale bio-molecular interaction. This information enables researchers to study the behavior of various kinds of biomolecules, particularly proteins. Not only that, this model provides information on the molecular affinity, whether the streptavidin molecules were well-adsorbed on the surface of the waveguide, or whether the under test antibodies were properly linked to the streptavidin.

## Conclusion

We have proposed an all-silicon platform for biomolecular on-chip spectroscopy using sub-THz frequencies. This device is capable of characterizing low-loss biosamples because of its unique and phenomenal features. Extremely low-loss waveguide, enhanced interaction of wave and sample through the longer length of the waveguide, and ease of fabrications are few advantages of this platform. Additionally, to the best of our knowledge, we have used the thin-film model for the first time to model the biomolecules in on-chip spectroscopy. The proposed model can be used to electromagnetically simulate the bioparticles more accurately, without incurring huge computational efforts. Utilizing the proposed platform in conjunction with the thin-film model enables low-loss, compact, and integrated devices for a wide range of applications in the field of spectroscopy and real-time biosensing.

## Data Availability

The datasets used and/or analysed during the current study available from the corresponding author on reasonable request.
